# A Storm of Polyserositis: Unravelling Multisystem Effusions in Hypereosinophilic Syndrome

**DOI:** 10.7759/cureus.74170

**Published:** 2024-11-21

**Authors:** Ahmed Fadel, Yasser Ahmed

**Affiliations:** 1 Respiratory Medicine, Dartford and Gravesham NHS Trust, Dartford, GBR

**Keywords:** community-acquired pneumonia mimic, corticosteroid therapy, diagnostic challenges, differential diagnosis, idiopathic hypereosinophilic syndrome, multidisciplinary management, pericardial effusion with cardiac tamponade, peripheral eosinophilia, polyserositis, rare cause of pleural effusion

## Abstract

Idiopathic hypereosinophilic syndrome (i-HES) is a rare disorder characterized by persistent eosinophilia and organ involvement, without an identifiable secondary cause. This case report describes a 42-year-old man with no significant medical history, who initially presented with symptoms resembling community-acquired pneumonia, including fever, shortness of breath, and pleuritic chest pain. Despite antibiotic treatment, his condition rapidly progressed to polyserositis, characterized by bilateral pleural effusions, pericardial tamponade, and ascites. Extensive diagnostic evaluation excluded the secondary causes of hypereosinophilia, leading to a final diagnosis of i-HES. Prompt initiation of high-dose corticosteroids resulted in dramatic clinical improvement, with resolution of the fever, pleural effusion, and pericardial effusion. This case highlights the diagnostic challenges associated with the atypical presentations of i-HES and emphasizes the importance of early recognition, comprehensive evaluation, and appropriate corticosteroid therapy. It also raises awareness of the potential for i-HES to present with life-threatening complications, underscoring the need for multidisciplinary management.

## Introduction

Hypereosinophilic syndrome (HES) is a heterogeneous group of disorders defined by persistent eosinophilia (absolute eosinophil count >1.5 × 10⁹/L) and evidence of organ involvement or dysfunction due to eosinophilic infiltration. Idiopathic hypereosinophilic syndrome (i-HES) refers to cases in which no secondary cause of eosinophilia such as infection, malignancy, or autoimmune disease can be identified. The condition is rare, with an estimated incidence of 0.16-0.36 per 100,000 and a prevalence of 0.36-6.3 per 100,000. i-HES can manifest with a wide range of clinical symptoms, reflecting the extent and severity of organ involvement, which may include the dermatological, pulmonary, gastrointestinal, cardiac, and neurological systems. This case report describes a 42-year-old man who presented with symptoms initially suggestive of community-acquired pneumonia but was subsequently found to have i-HES with life-threatening complications, including polyserositis and cardiac tamponade. This report illustrates the challenges in diagnosing i-HES, particularly in cases with atypical presentations, and emphasizes the need for a comprehensive diagnostic approach and prompt therapeutic intervention. It also highlights the role of corticosteroids as the mainstay of treatment and their potential for rapid clinical improvement with timely management.

## Case presentation

A 42-year-old man with no significant medical or family history and no prescription or over-the-counter medications presented with a one-week history of fever, shortness of breath, and pleuritic chest pain. He reported no recent travel or known exposure to tuberculosis (TB). Physical examination revealed decreased air entry and a few crackles in the right lung. The patient’s vital signs were stable except for a fever of 38°C.

**Figure 1:**
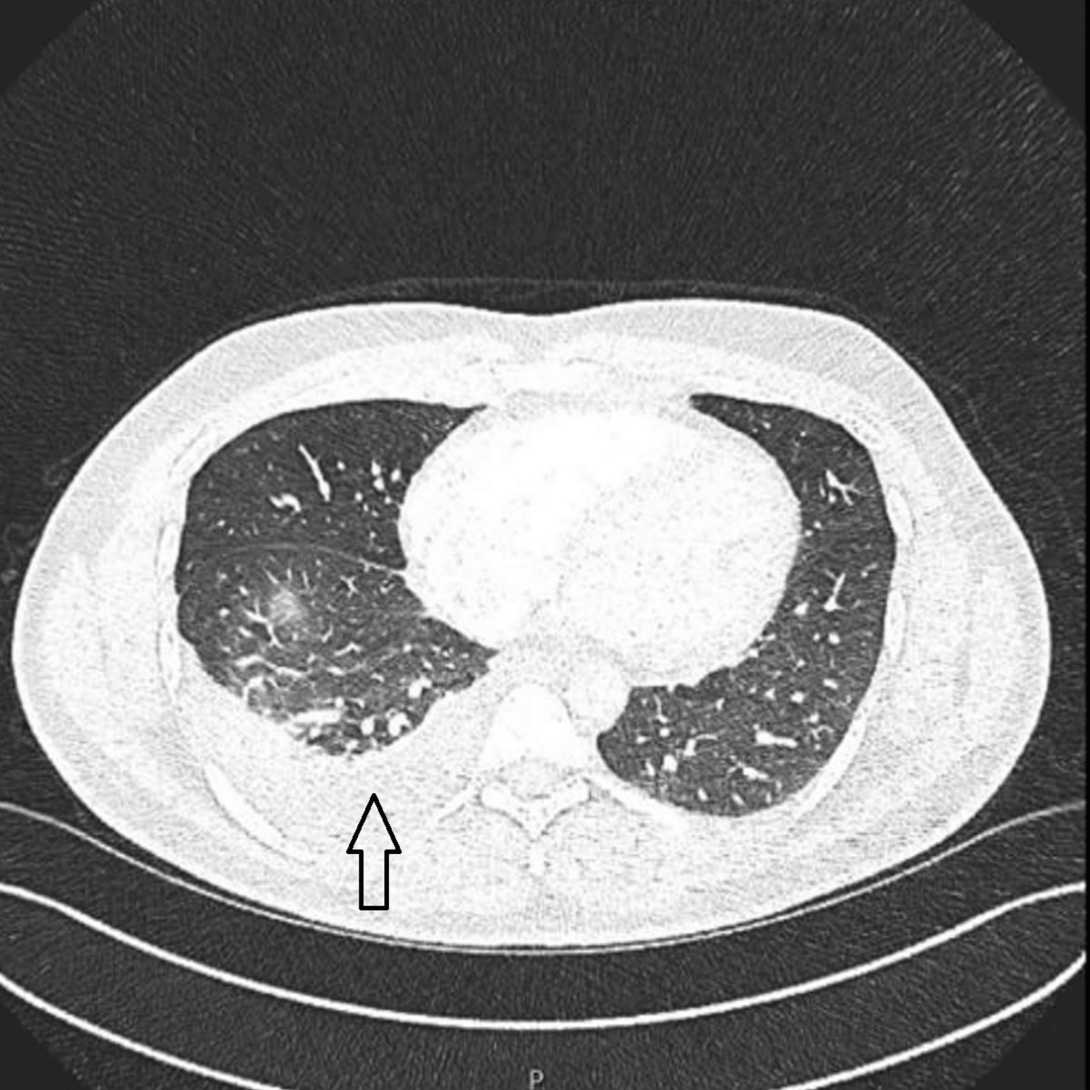
CT pulmonary angiography showing no pulmonary embolism but showing small right-sided pleural effusion and right lower lobe consolidations.

Blood tests showed a white blood cell (WBC) count of 9.8 × 10⁹/L (normal range: 4-11 × 10⁹/L), an elevated absolute eosinophil count of 0.9 × 10⁹/L (normal range: 0.0-0.4 × 10⁹/L), an increased C-reactive protein (CRP) level of 32.3 mg/L (normal range: <5 mg/L), and a D-dimer level of 799 ng/mL (normal range: <500 ng/mL). Given the elevated D-dimer level, CT pulmonary angiography (CTPA) was performed to rule out pulmonary embolism, which was subsequently excluded. However, the scan revealed a small right-sided pleural effusion and consolidation in the right lower lobe (Figure 1).

The patient was initially diagnosed with community-acquired pneumonia and discharged after a seven-day course of oral levofloxacin. However, his symptoms persisted, and he returned a week later with worsening breathlessness and fever.

Upon readmission, blood tests revealed a WBC count of 11.0 × 10⁹/L (normal range: 4-11 × 10⁹/L), an elevated absolute eosinophil count of 1.4 × 10⁹/L (normal range: 0.0-0.4 × 10⁹/L), and a markedly increased CRP level of 105 mg/L (normal range: <5 mg/L). Diagnostic pleural aspiration showed dark yellowish fluid with a pH of 7.42. Biochemical analysis confirmed exudative pleural effusion, indicated by a lactate dehydrogenase (LDH) level of 1115 U/L (normal range: <200 U/L) and a protein concentration of 56 g/L (normal range: <30 g/L).

The patient was initially administered parenteral amoxicillin and clarithromycin. However, owing to persistent fever and pleural effusion, the antibiotics were later changed to ceftazidime and vancomycin.

Pleural fluid cultures were negative. Given the concern for possible TB-associated pleural effusion, sputum samples were collected for three consecutive days to test for acid-fast bacilli (AFB). Additionally, pleural fluid was sent for interferon-gamma release Aassay (IGRA) and adenosine deaminase (ADA) testing. The combined negative results of these tests have a high sensitivity for excluding TB-related pleural effusion [1,2].

Unfortunately, the patient was not stable enough to undergo thoracoscopy for pleural biopsy, which remains the gold standard for diagnosing TB pleuritis. Both the IGRA and ADA tests returned negative results, making the possibility of TB as the cause of pleural effusion less likely. Later, the pleural fluid culture returned negative after 56 days of incubation in the liquid culture.

Pleural fluid cytology revealed eosinophil-rich fluid with no detected malignant cells. Multiple blood and sputum cultures tested negative for microbial growth. Bronchoalveolar lavage was not considered because the patient was not sufficiently stable for bronchoscopy. The patient’s oxygen requirement increased to an FiO_2_ of 40%, but mechanical intubation was not indicated.

Detailed examination did not reveal any vascular or immunological phenomena suggestive of infective endocarditis. The patient underwent transthoracic echocardiography, which revealed a preserved ejection fraction with no evidence of vegetation or pericardial effusion. A transesophageal echocardiogram was not deemed necessary because the likelihood of infective endocarditis was very low based on Duke’s criteria.

**Figure 2:**
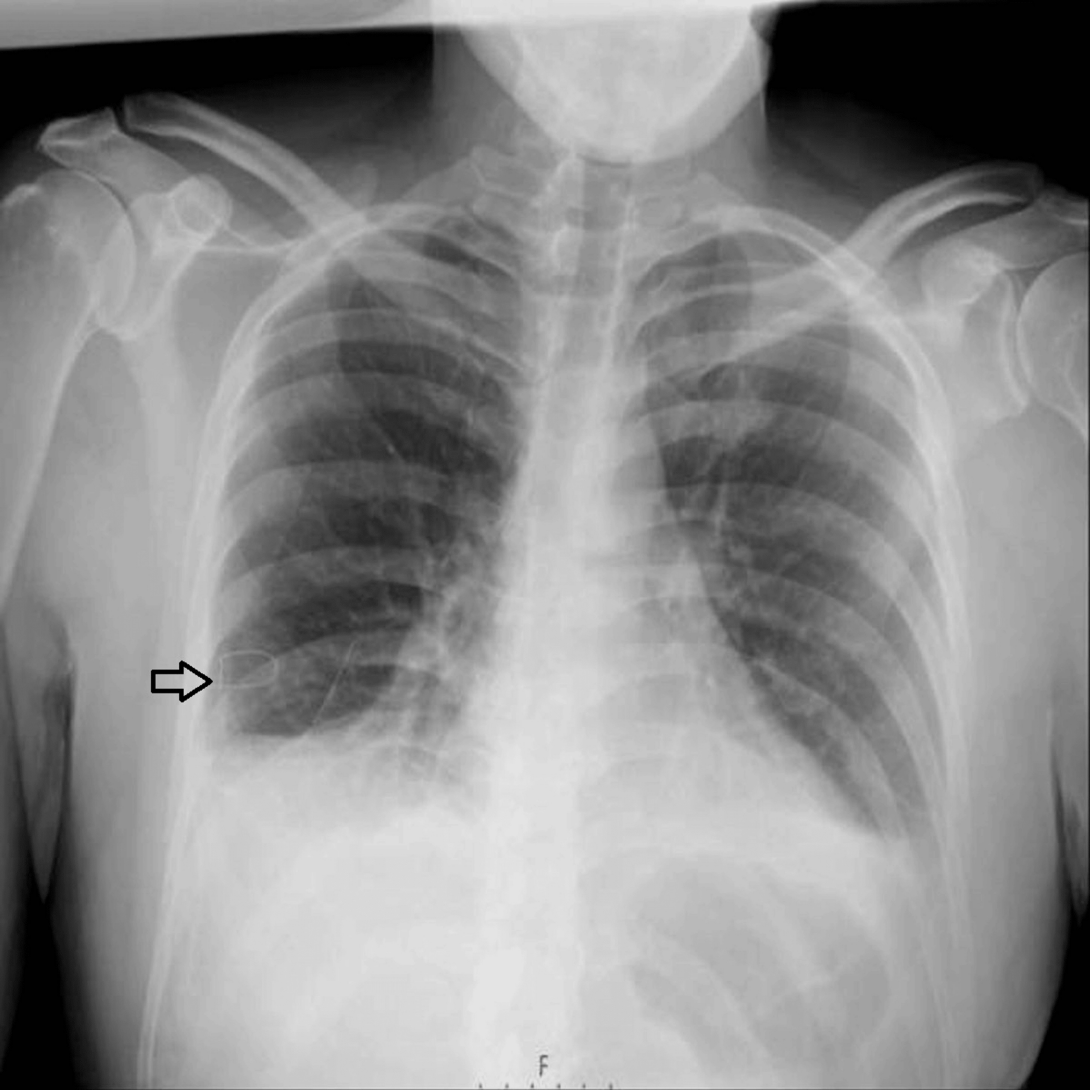
Chest X-ray after the insertion of the right-sided chest drain.

Owing to progressive breathlessness caused by the enlarging right-sided pleural effusion, a chest drain was inserted and later removed once the drainage ceased (Figure 2).

During this period, the patient’s absolute eosinophil count continued to increase, reaching over 1.5 × 10⁹/L, which exceeds the threshold for HES. Other routine blood tests showed normal renal function, with mildly elevated liver function, including an alanine aminotransferase level of 76 U/L (normal range: 7-56 U/L).

A detailed drug history, along with investigations for infectious, autoimmune, and parasitic causes, including stool examination and serology for parasites, such as *Paragonimus westermani*, failed to identify any secondary cause of hypereosinophilia (Table 1).

**Table 1:** Detailed infectious and autoimmune screen results. BNP: B-type natriuretic peptide; ANCA: anti-neutrophil cytoplasmic antibodies; EBV: Epstein–Barr virus; CMV: cytomegalovirus; HIV: human immunodeficiency virus; CCP: cyclic citrullinated peptide; ENA: extractable nuclear antigen; ANA: antinuclear antibody; ACE: angiotensin-converting enzyme

Test	Result	Comment	Reference range
Vitamin B12	171 ng/L	Normal	160–950 ng/L
IgG	8.9 g/L	Normal	7.0–16.0 g/L
IgA	3.81 g/L	Normal	0.85–4.5 g/L
IgM	0.89 g/L	Normal	0.5–2.0 g/L
IgE	277 KU/L	Elevated	<100 KU/L
Troponin	3.5 ng/L	Normal	<14 ng/L
Plasma BNP	25.0 pg/L	Normal	<100 pg/L
Rheumatoid Factor	2 IU/L	Normal	<14 IU/L
ANCA screen	Negative	Normal	Negative
Parasitology screen	Negative	Normal	Negative
C3	1.91 g/L	Mildly elevated	0.9–1.8 g/L
C4	0.32 g/L	Normal	0.1–0.4 g/L
Hepatitis C antibodies	Negative	Normal	Negative
CMV antibodies	IgG positive, IgM negative	Normal	IgG positive, IgM negative indicates past infection
HIV antibodies	Negative	Normal	Negative
Tryptase	5.5 ng/L	Normal	<11.4 ng/L
Anti-CCP antibodies	2 U/L	Normal	<20 U/L
Anti-dsDNA antibodies	3 IU/L	Normal	<15 IU/L
ENA antibody screen	Negative	Normal	Negative
ANA antibodies	Negative	Normal	Negative
Serum ACE level	17 U/L	Normal	8–52 U/L

**Figure 3:**
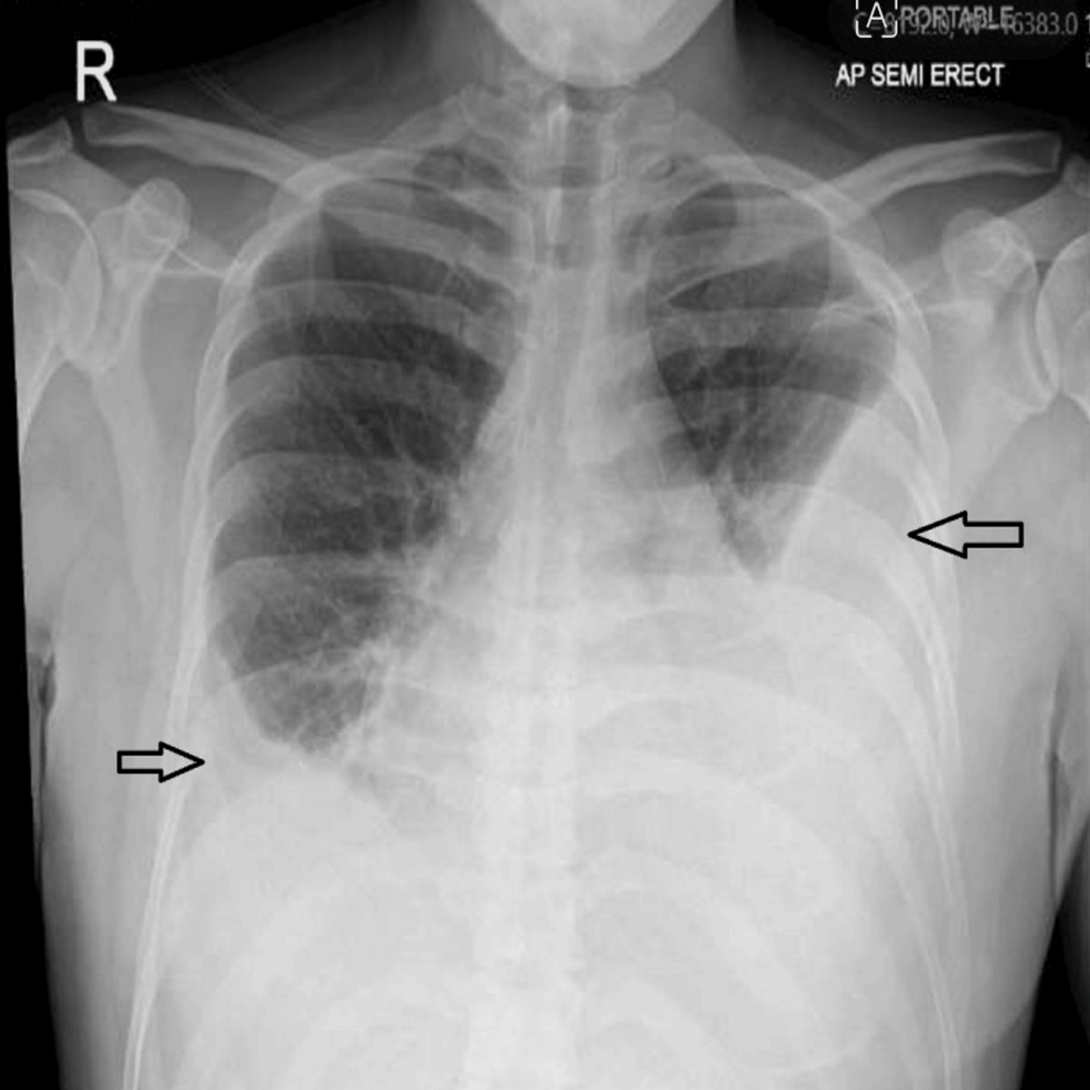
Chest X-ray showing new large left-sided pleural effusion and recurrence of right-sided pleural effusion.

During laboratory evaluation for hypereosinophilia and pleural effusion, the patient developed worsening shortness of breath, leading to a repeat chest X-ray, which showed a large left-sided pleural effusion (Figure 3).

**Figure 4:**
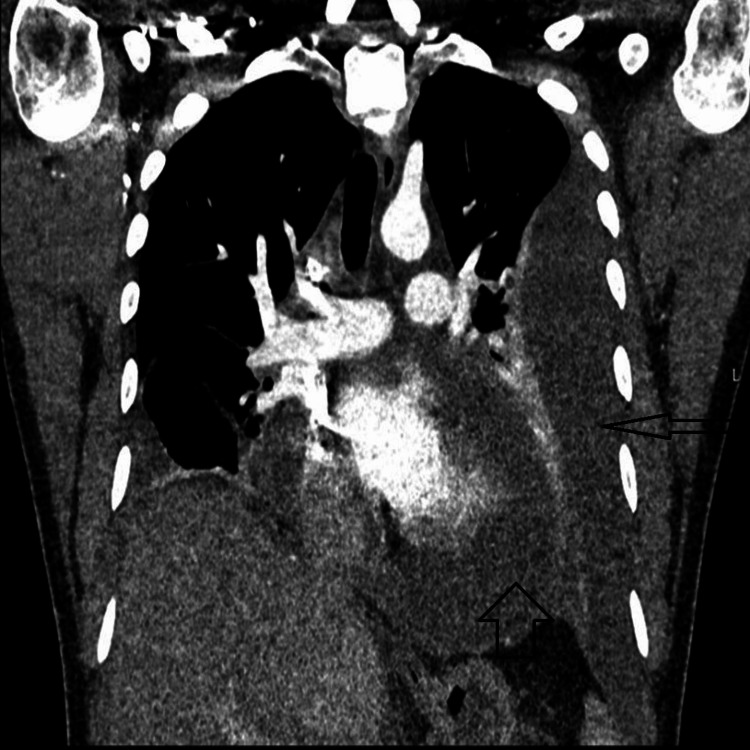
Moderate right-sided and large left-sided pleural effusion with large pericardial effusion and moderate ascites.

A subsequent CT scan of the chest, abdomen, and pelvis confirmed a large left-sided pleural effusion, moderate right-sided pleural effusion, large pericardial effusion, and new moderate ascites, with no evidence of splenomegaly or lymphadenopathy (Figure 4).

Repeat echocardiography revealed a new pericardial effusion measuring over 2 cm, with signs of late diastolic collapse of the right ventricle, indicating early cardiac tamponade. Due to the patient’s hemodynamic instability and persistent tachycardia, he was transferred to the cardiac care unit, where a pericardial drain was placed, removing 600 mL of fluid. Analysis of the fluid showed that it was rich in eosinophils, with no malignant cells detected, and negative culture results. The drain was removed two days later, once fluid drainage had ceased.

**Figure 5:**
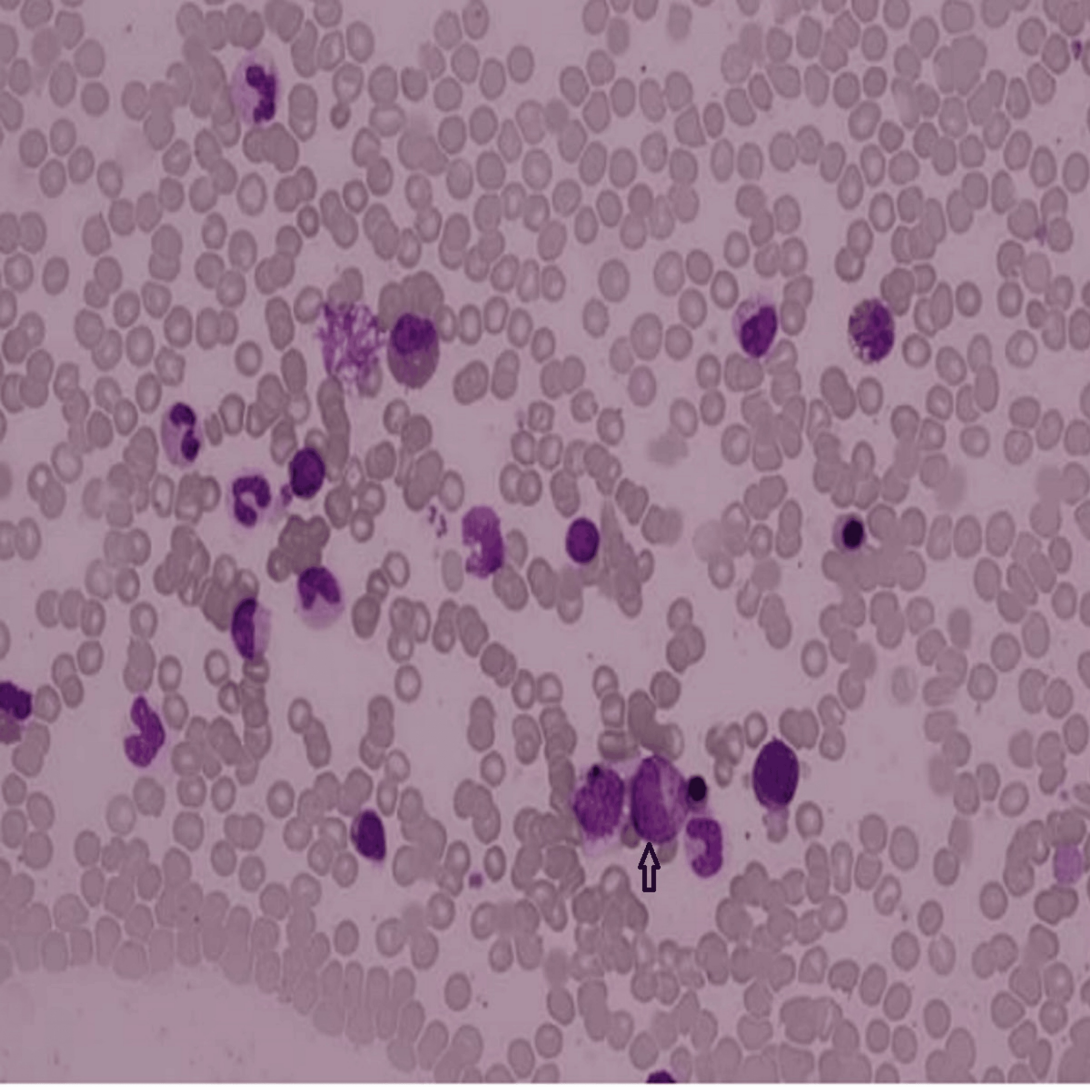
Bone marrow aspirate: normocellular particles with increased eosinophils.

With a strong clinical suspicion of HES underlying his symptoms and given the life-threatening nature of pericardial effusions, prompt initiation of treatment is crucial. High-dose prednisolone (1 mg/kg) was administered, and bone marrow aspiration was performed concurrently. Aspiration was prioritized before starting steroid therapy, as corticosteroids could affect the diagnostic yield, particularly in cases in which a lymphocytic variant of HES might be present which has lymphoma-like pathology [3] (Figure 5, Table 2).

**Table 2:** Bone marrow aspiration showing no malignant cells but revealing increased eosinophils.

Investigation	Result
*BCR:ABL1*, *FIP1L1:PDGFRA*, *PDGFRB*, *JAK2*, *FGFR1*, and *KIT*	Negative
Bone marrow immunophenotyping	CD34+ myeloid progenitors accounted for 0.4% of the total nucleated cell count with a CD117+, HLADR+, CD33+, and CD13+ phenotype. No clonal B-cells were identified. Negative for the lymphocytic variant of hypereosinophilic syndrome
Bone marrow microscopy	Increased eosinophil count. No evidence of lymphoma or myeloid pathology

**Figure 6:**
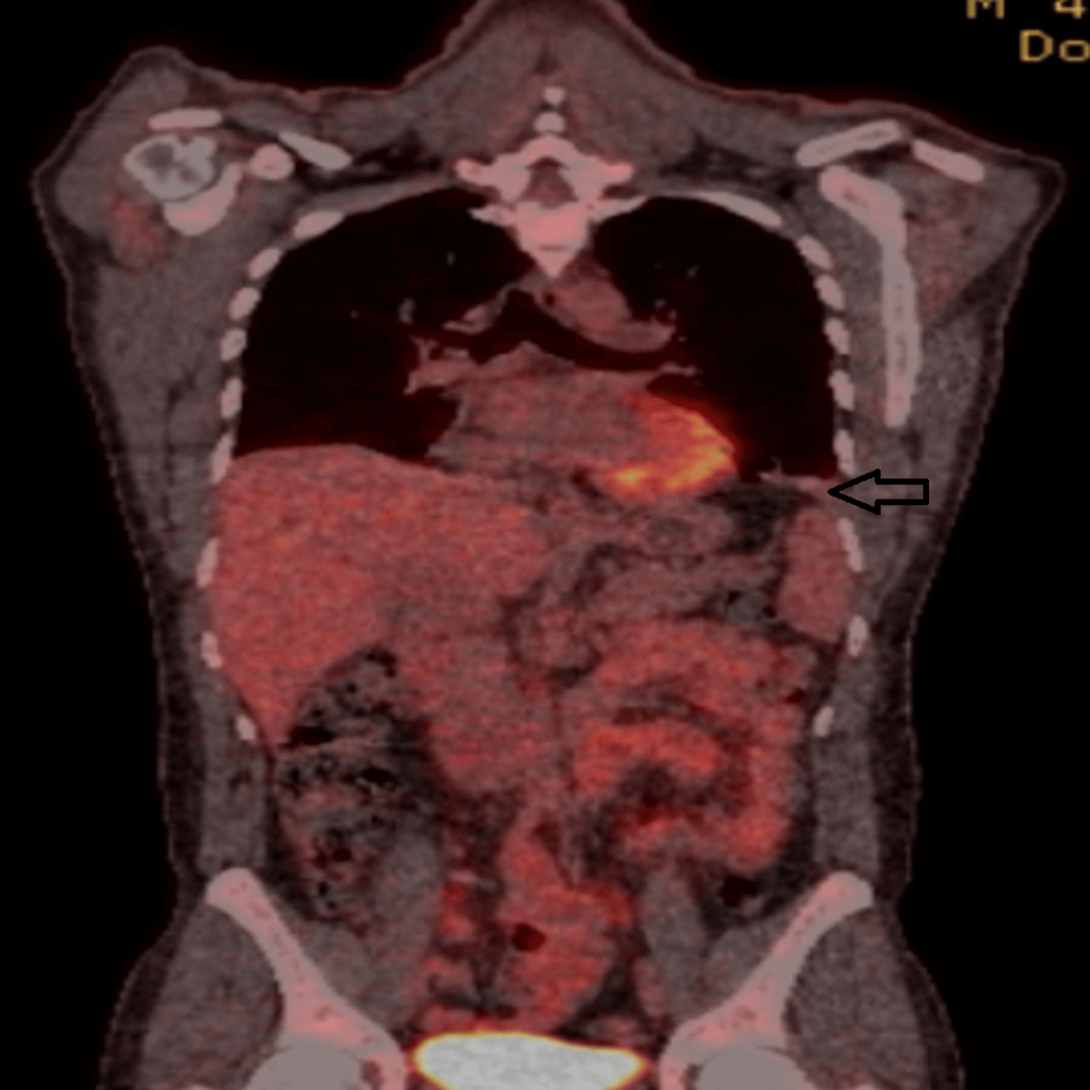
Positron emission tomography revealing low-grade to mildly 18F-fluorodeoxyglucose-avid left-sided pleural effusion with no splenomegaly or lymphadenopathy.

The patient experienced dramatic recovery following the initiation of prednisolone, with the fever resolving and the pleural effusion gradually decreasing without the need for additional drainage, as confirmed by follow-up chest radiography. Positron emission tomography (PET) revealed low-grade to mildly 18F-fluorodeoxyglucose (FDG)-avid left-sided pleural effusion, with no other abnormalities detected (Figure 6).

**Figure 7:**
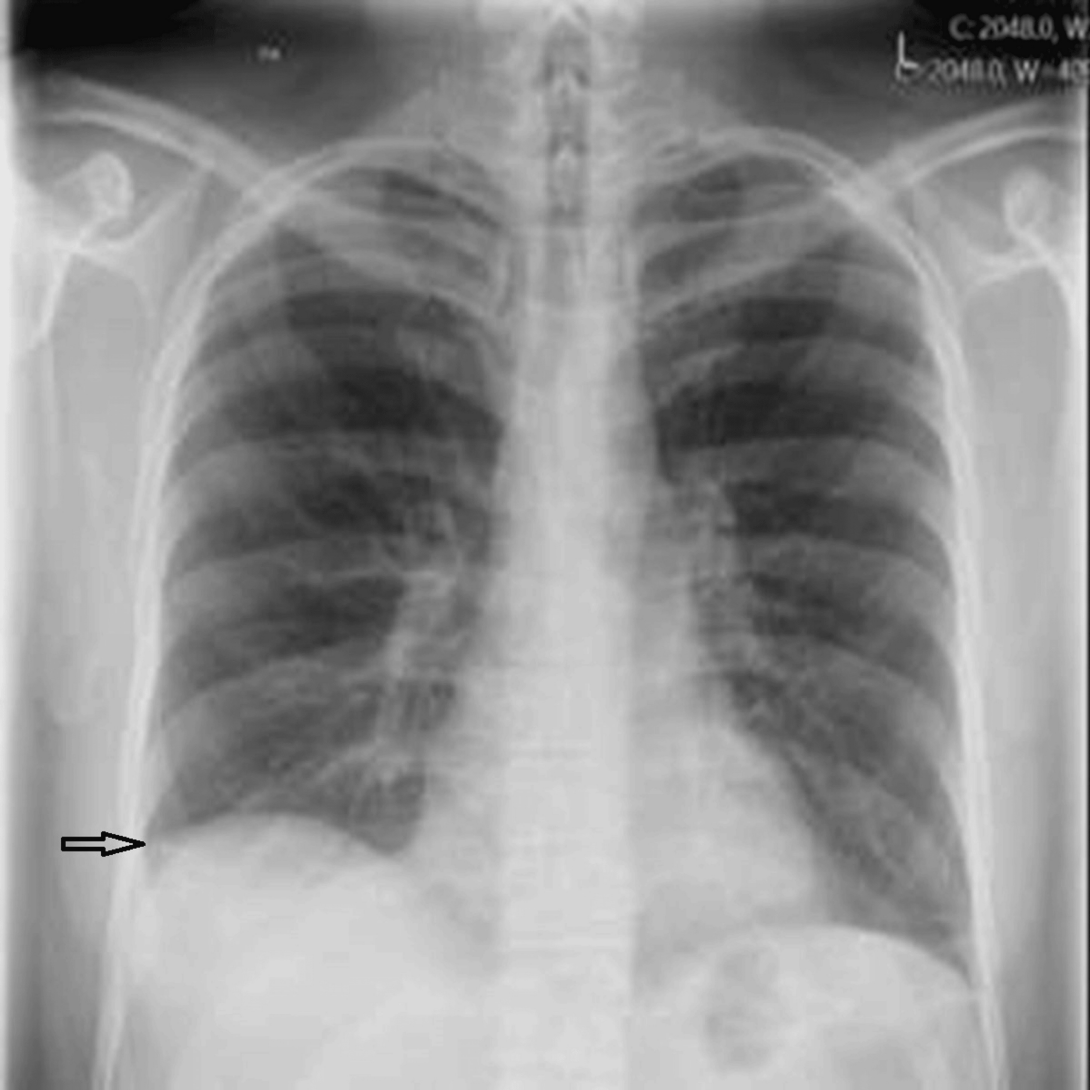
Chest X-ray showing near-complete resolution of left-sided pleural effusion without drainage and minimal right-sided pleural effusion without drainage.

A final diagnosis of iHES was made after excluding secondary and hematological causes of hypereosinophilia. The patient was discharged on a carefully planned, slow-tapering regimen of oral prednisolone. A follow-up chest radiograph two months later, along with repeat echocardiography, confirmed that there was no recurrence of the pleural (Figure 7) or pericardial effusion.

**Figure 8:**
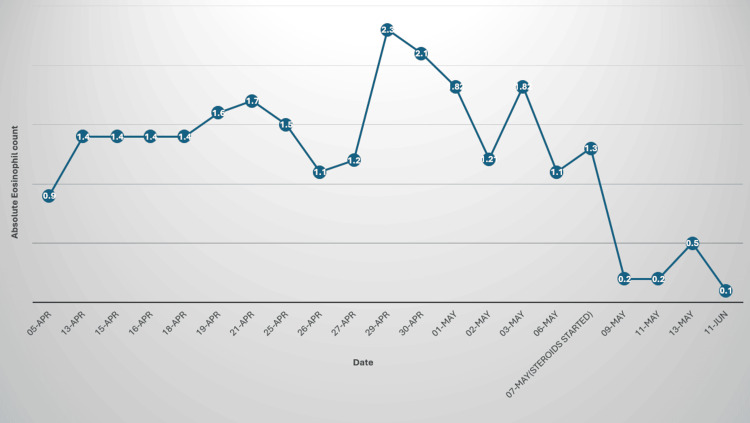
Trend of eosinophil count before and after steroids.

Steroid treatment led to a rapid decrease in the patient’s absolute eosinophil count, which progressively normalized over the course of follow-up. Clinically, the patient demonstrated significant improvement, with the fever resolving and pleural effusion markedly reduced. Subsequent imaging confirmed the complete resolution of the effusions, and the patient remained asymptomatic despite ongoing prednisolone tapering (Figure 8).

## Discussion

This case highlights an unusual presentation of i-HES, which initially manifested as a small right-sided pleural effusion and consolidation suggestive of community-acquired pneumonia, potentially complicated by parapneumonic effusion, without any initial evidence of pericardial effusion. The patient’s condition rapidly progressed, leading to polyserositis, characterized by bilateral pleural effusions, moderate ascites, and pericardial tamponade, necessitating drainage.

**Figure 9:**
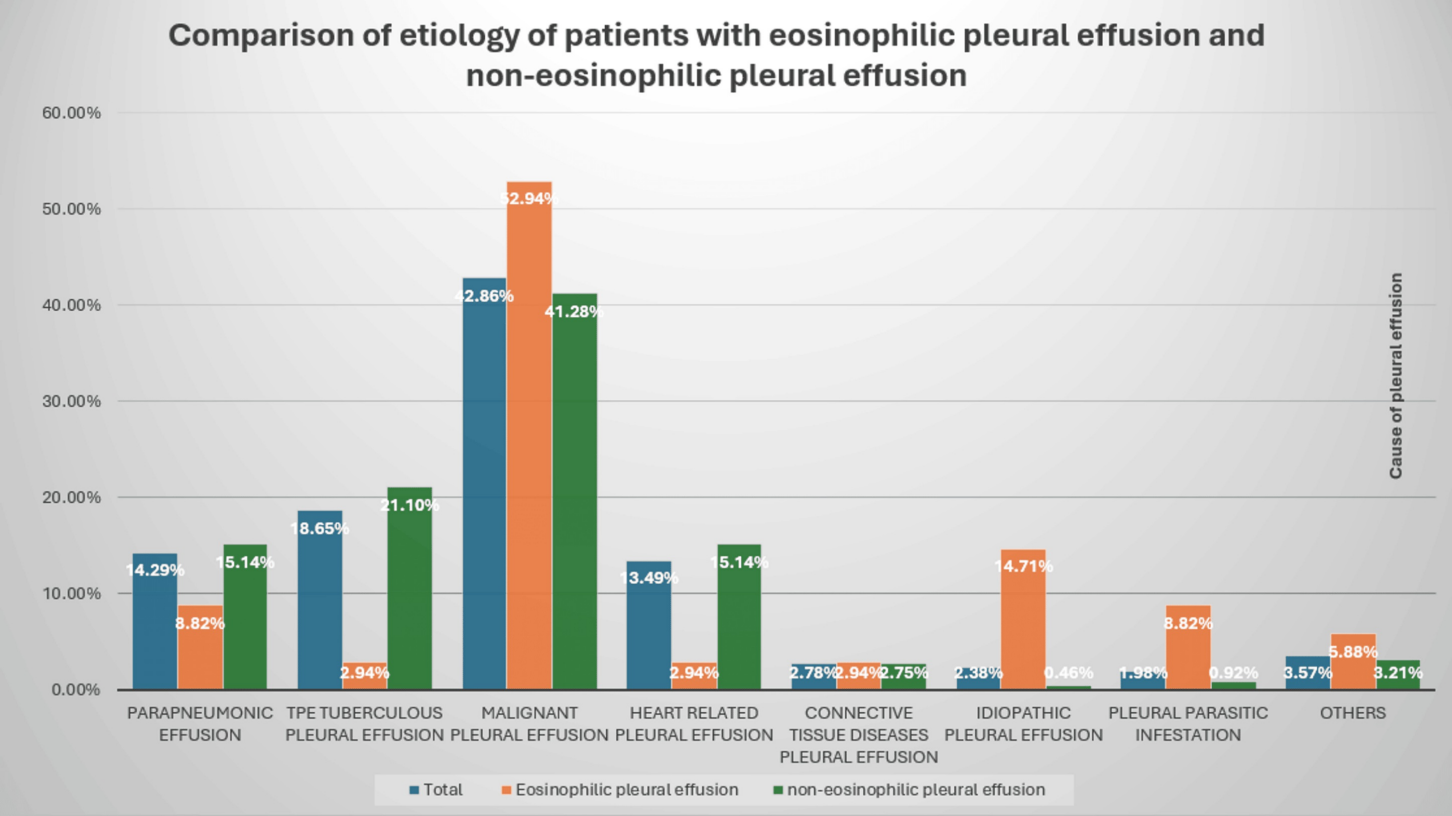
Comparison of the etiology of patients with eosinophilic and non-eosinophilic pleural effusion. Adapted from Li et al. [5].

Initially, the patient presented with a right-sided pleural effusion and an elevated eosinophil count, though it was below the diagnostic threshold of 1.5 × 10⁹/L typically used for diagnosing HES. Consequently, other differential diagnoses were also explored. In two retrospective studies, eosinophilic pleural effusion was identified in 7.2% and 13.49% of all pleural effusion cases. In both studies, the most common cause of eosinophilic pleural effusion was malignancy, accounting for 34.8% in the first study, followed by infectious causes (19.2%), unknown origin (14.1%), post-traumatic causes (8.9%), and miscellaneous conditions (23.0%). The second study reported malignancy as the leading cause (52.94%), followed by i-HES (14.71%), parasitic infections (8.82%), pneumonia (8.82%), and others (14.71%) [4,5] (Figure 9).

Based on the patient’s history and clinical presentation, the right-sided pleural effusion was initially thought to be parapneumonic in origin. However, repeated negative results from blood and pleural fluid cultures, coupled with a lack of response to broad-spectrum antibiotics, made this diagnosis unlikely. Tuberculous pleural effusion was also considered, but multiple tests, including pleural fluid IGRA and ADA assays [1,2], repeated TB smears and cultures from pleural fluid, sputum TB cultures, and GeneXpert analysis, were all negative, effectively ruling out TB. Malignancy was further excluded, as pleural fluid cytology showed no malignant cells, and imaging findings were normal, with no evidence of neoplastic disease. Given the increasing eosinophil count, multisystemic causes were subsequently explored in the differential diagnosis.

Hypereosinophilia is defined as an eosinophil count of 1.5 × 10^9^/L or greater, persisting for at least six months. HES is characterized by an elevated eosinophil count and evidence of organ involvement or dysfunction attributable to eosinophilia [6-8]. However, the suggested revised classifications have shortened the mandated time for diagnosis to ≥1 month, allowing for earlier identification and management of the condition. Importantly, treatment can be initiated at any time if there is evidence of organ involvement, without waiting to fulfill the full criteria of persistent eosinophilia for six months [6,8].

Definition of eosinophilia based on the suggested revised classification [8,9]: Eosinophilia: 0.5-1.5 × 10^9^/L; hypereosinophilia: >1.5 eosinophils × 10^9^/L blood on two tests ≥1 month apart and/or tissue hypereosinophilia; hypereosinophilic syndrome: diagnosis of hypereosinophilia >1.5 eosinophils × 10^9^/L blood on two tests ≥1 month apart and/or tissue HE, associated with end-organ damage secondary to the hyperesinophilia [8] (Table 3).

**Table 3:** Classification of hypereosinophilia.

Type	Description
Clonal hypereosinophilia (previously a myeloid variant of hypereosinophilia/hypereosinophilic syndrome)	Hematopoietic neoplasm associated with abnormal proliferation of eosinophilic precursors, e.g., cytogenetics for mutations *BCR:ABL1*, *FIP1L1-PDGFRA*, *PDGFRB*, *JAK2*, *FGFR1*, and *KIT*. Failure to detect the *FIP1L1:PDGFRA* fusion gene does not necessarily rule out the diagnosis of clonal hypereosinophilia
Reactive hypereosinophilia	Polyclonal expansion of eosinophils, secondary to abnormal production of high amounts of eosinophilopoietic cytokines. Lymphocytic hypereosinophilic syndrome and idiopathic hypereosinophilic syndrome are included in this group
Familial hypereosinophilia	Multiple unexplained hypereosinophilia cases found in certain families

**Figure 10:**
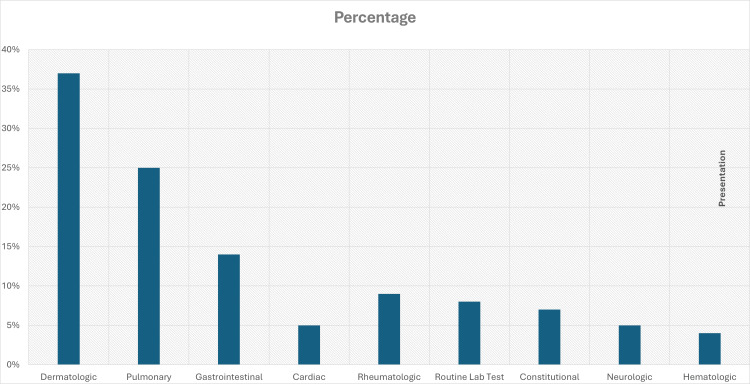
Presentations of Idiopathic hypereosinophilic syndrome. Adapted from Ogbogu et al. [10].

A 2009 multicenter retrospective study documented that the most common manifestations of hypereosinophilia were dermatological (37%) and pulmonary (25%) (Figure 10) [10].

**Figure 11:**
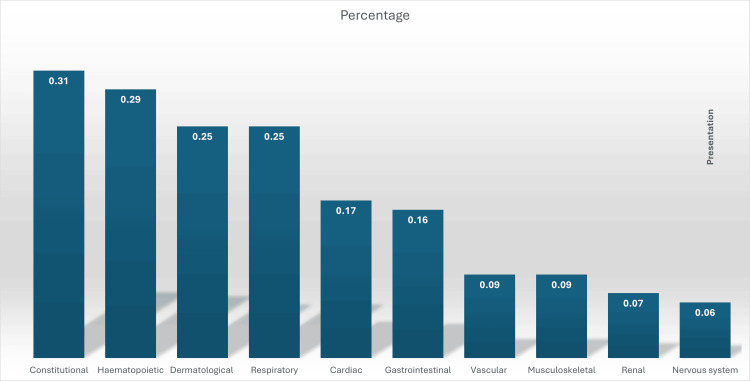
Presentations of idiopathic hypereosinophilic (iHES) syndrome according to a nationwide survey in Japan on iHES. Adapted from Honda et al. [11].

However, these previous findings contrast with a recently published nationwide survey in Japan on iHES, in which constitutional manifestations were the most common presenting symptoms with a median involvement of three organs per patient (Figure 11) [11].

In HES, organ involvement is characterized by eosinophil-mediated damage or dysfunction in one or more organs. The histological or cytological criteria for diagnosing organ involvement include eosinophilic infiltration of the bone marrow exceeding 20% or significant eosinophilic infiltration of tissues, as assessed by a pathologist [8].

Once HES is suspected, a comprehensive workup is essential to confirm the diagnosis and evaluate the extent of organ involvement. This workup should include a full blood count, blood film examination, liver and renal function tests, and additional investigations tailored to the suspected organ involvement. Early and accurate identification of organ involvement is crucial for guiding appropriate management and improving patient outcomes [6,12] (Table 4).

**Table 4:** Investigations done in suspected cases of HES. ESR: erythrocyte sedimentation rate; CRP: C-reactive protein; PCR: polymerase chain reaction; FISH: fluorescence in situ hybridization; PET: positron emission tomography; HES: hypereosinophilic syndrome

Full blood count, blood film examination, liver and renal function tests, ESR, CRP, and vitamin B12	All patients
BCR-ABL1, PDGFRB, JAK2, FGFR1, and KIT mutations by PCR and FISH	Patients with unexplained eosinophil count ≥1.5 × 10^9^/L
Serum tryptase	Patients with unexplained eosinophil count ≥1.5 × 10^9^/L
Bone marrow aspirate, trephine biopsy, and cytogenetic analysis	Negative FISH or RT-PCR for *FIP1L1*-*PDGFRA*
Pulmonary function testing	In the case of end-organ pulmonary involvement
Chest/Abdomen/Pelvis CT	In the case of the chest (end-organ affection) to assess for lymphadenopathy, splenomegaly, and occult neoplasms
Serum troponin and echocardiogram	In case of end-organ cardiac involvement Grade 1C
Immunoglobulin levels, IgE, and serum protein electrophoresis	All cases
Lymphocyte immunophenotyping	In case of suspected lymphocytic HES
PET scan	In case of suspected lymphoma or occult malignancy

In this case, the patient showed negative fluorescence in situ hybridization results for *BCR*, *FIP1L1*, *PDGFRB*, *JAK2*, *FGFR1*, and *KIT*. Detailed genetic studies and bone marrow trephine biopsy microscopy ruled out a myeloproliferative HES. Additionally, negative serum tryptase levels and immunophenotyping excluded systemic mastocytosis, chronic eosinophilic leukemia, and the lymphocytic variant of HES. Imaging studies, including a PET scan, normal serum protein electrophoresis, and a CT scan of the chest, abdomen, and pelvis, showed no evidence of occult malignancy. Following the exclusion of secondary causes, a diagnosis of i-HES was established, with elevated IgE levels suggesting a favorable response to corticosteroids.

Given the severity of the condition and the presence of organ involvement, high-dose corticosteroid therapy with prednisolone (1 mg/kg) was initiated. A pericardial drain was also placed to manage tamponade caused by pericardial effusion. The patient exhibited dramatic improvement after the start of corticosteroid treatment, with resolution of fever and gradual reduction of the pleural effusion. Pericardial effusion did not recur after the drain was removed.

Polyserositis is a rare manifestation of i-HES [13], with reports in the literature indicating the occurrence of pericardial effusion in approximately 4% of cases [11,14], pleural effusion in 4% [11,15,16], and ascites in 2% of cases [11]. HES is characterized by the persistent overproduction of eosinophils, leading to tissue infiltration and damage. Although the exact pathophysiology remains unclear, it is believed to involve both clonal and reactive mechanisms. In this case, the absence of clonal markers, such as the *FIP1L1*-*PDGFRA* fusion gene, and the lack of identifiable secondary causes, including parasitic infection or drug reaction, supported the diagnosis of iHES.

The rarity of HES makes its incidence and prevalence uncertain. However, a 2010 study estimated the incidence to be 0.16-0.36 per 100,000 and the prevalence to be 0.36-6.3 per 100,000 [17]. iHES is a diagnosis of exclusion, requiring a thorough evaluation to rule out other potential causes. Corticosteroids are the cornerstone of treatment, typically starting at a dose of 0.5-1 mg/kg/day of prednisolone, followed by gradual tapering over two to three months. When there is a history suggestive of potential strongyloidiasis, empirical ivermectin should be administered along with steroids to prevent *Strongyloides* hyperinfection syndrome [6].

Several factors are associated with a worse five-year prognosis in iHES, including age >50 years, hemoglobin levels <12 g/dL, activated partial thromboplastin time greater than 34 seconds, dyspnea, thrombotic tendency, renal failure, cardiac involvement, thrombocytopenia, and splenomegaly, which is also linked to a poorer two-year survival rate [11]. If the response to corticosteroids is inadequate, if a high maintenance dose is required, or if the patient experiences side effects, alternative treatments such as imatinib, immunomodulatory agents such as interferon-alpha, myelosuppressive drugs such as hydroxyurea, or biologics such as mepolizumab may be considered [6,18]. The anti-CD52 monoclonal antibody alemtuzumab is an option for severe iHES that is unresponsive to other therapies, particularly in cases involving cardiac or neurological complications [6].

In this case, the rapid decline in eosinophil count following corticosteroid therapy was indicative of a good prognosis. Nevertheless, regular follow-up is essential to monitor potential complications. It has also been noted that iHES can sometimes be reclassified as myeloproliferative or lymphocytic HES during long-term follow-up [19].

## Conclusions

This case demonstrates a rare presentation of i-HES that initially resembled pneumonia but rapidly progressed to life-threatening polyserositis with pericardial tamponade. The diagnostic challenges, including an atypical presentation and the need to exclude secondary causes, contributed to the delay in initiating corticosteroid treatment. Early administration of corticosteroids may have prevented severe complications, underscoring the importance of prompt recognition and intervention in cases of i-HES. The patient’s dramatic improvement following high-dose prednisolone reaffirmed the role of corticosteroids as the cornerstone of i-HES therapy. This case also highlights that eosinophil count may not consistently correlate with the severity of the disease, suggesting a need for refinement of the diagnostic criteria for HES. Comprehensive diagnostic workup, multidisciplinary management, and adherence to evidence-based guidelines are crucial for optimizing patient outcomes. Regular follow-up is essential to monitor for potential complications and reassess the diagnosis as i-HES can evolve into other variants over time. This report contributes to the understanding of i-HES by documenting a rare presentation and advocating for the refinement of diagnostic and therapeutic approaches.
